# The impact of inhalation anesthetics on early postoperative cognitive function and recovery characteristics in Down syndrome patients: a randomized, double – blind study

**DOI:** 10.1186/s12871-021-01447-x

**Published:** 2021-09-17

**Authors:** Emmanouil Gkliatis, Alexandros Makris, Chryssoula Staikou

**Affiliations:** 1 Department of Anesthesiology, Asklepieion Hospital of Voula, Vas. Pavlou 1, 16673 Athens, Greece; 2 Aretaieion Hospital, Medical School, National and Kapodistrian University of Athens, Greece, Athens, Greece

**Keywords:** Sevoflurane, Desflurane, Down syndrome, Anesthesia

## Abstract

**Background:**

Down syndrome (DS) is associated with intellectual disability. DS patients may be unable to cooperate and often require general anesthesia even for minor surgeries. Rapid recovery significantly contributes to fast-tracking. This prospective randomized, double - blind study investigates the impact of desflurane and sevoflurane on recovery and early postoperative cognitive function of these patients.

**Methods:**

Forty-four patients undergoing dental surgery, were randomized to receive desflurane (DES-group) or sevoflurane (SEVO-group) for anesthesia maintenance. The primary outcome was postoperative cognitive function (Prudhoe Cognitive Function Test, PCFT) at 90 min and 4 h postoperatively. Secondary outcome measures were the time between volatile discontinuation and spontaneous breath, eye opening, extubation, orientation and response to commands, time to achieve an Aldrete score ≥ 9 in the Post-anesthesia Care Unit and time to fulfill discharge criteria (Post Anesthetic Discharge Scoring System, PADSS).

**Results:**

At 90 min, PCFT scores significantly decreased from baseline in both groups. Nevertheless, at 4 h, in DES-group there was no significant change from baseline (*p* = 0.163), while in SEVO-group the decrease remained significant (*p* < 0.001). Desflurane was also found superior regarding recovery characteristics, such as time to eye opening (*p* = 0.021), spatial orientation (*p* = 0.004), response to commands (*p* = 0.004). Discharge criteria were met earlier in DES-group (*p* = 0.018 for Aldrete score / *p* < 0.001 for PADSS).

**Conclusions:**

Desflurane was found superior to sevoflurane in terms of faster recovery and better preserved postoperative cognitive function in DS patients undergoing dental surgery. We suggest that desflurane, as part of a multimodal anesthetic approach, could be a useful agent to enhance early discharge from hospital of ambulatory patients with intellectual disability.

**Trial registration:**

Registered with ClinicalTrials.gov (NCT02971254, principal investigator: E.G; November 2016).

## Background

Down syndrome (DS) is the most common chromosomal abnormality in humans, caused by the presence of a full or partial third copy of chromosome 21. It is associated with a variety of birth defects and diseases, including developmental, neurological and neurotransmitter alterations, intellectual disability (ID) and increased occurrence of Alzheimer’s disease. The improvement in medical care over the years has significantly increased the life expectancy of individuals with DS, and survival into adulthood is now reasonably expected [1].

The DS is the commonest genetic cause of ID that represents a constant and characteristic feature of the syndrome [2]. The degree of ID varies significantly among people with DS, ranging from mild to moderate or severe. However, most commonly it falls within the mild to moderate range [3]. These patients may be unable to comprehend the purpose of any medical intervention and it is difficult to cooperate even for minor surgical procedures. Within this context, dental treatment for DS patients with ID is often performed under day care general anesthesia.

On the other hand, anesthetic drugs may transiently impair patients’ cognitive function and mental status postoperatively, thus delaying their discharge from hospital, especially in day case surgery. Rapid recovery from anesthesia significantly contributes to fast-tracking through postoperative recovery steps and discharge, increases patient comfort and satisfaction, while reducing financial costs and nursing workload in ambulatory care settings [4].

Volatile anesthetics with low blood-gas partition coefficients, such as desflurane and sevoflurane, may be associated with less postoperative cognitive impairment and confusion. There are several studies comparing the two volatiles regarding their recovery characteristics and effects on postoperative cognition. Still, they are mainly focused on elderly population and present contradictory results, favoring none of the two or slightly favoring desflurane [5–14]. To our knowledge the present study is the first to examine the impact of the two most popular volatile anesthetics, namely desflurane and sevoflurane, on recovery and early postoperative cognitive function of patients with preexisting ID, specifically adult DS patients.

## Methods

The present randomized, double-blind, parallel-group study was approved by the Institutional Review Board of the Asklepieion Hospital of Voula (identification number: 8174/2016) and was registered with ClinicalTrials.gov (NCT02971254, principal investigator: E.G; Date of Registration: 22/11/2016). Inclusion criteria were Down syndrome patients scheduled for dental surgery, age above 14 years and American Society of Anesthesiologists (ASA) classification I - III. Exclusion criteria were severe visual or hearing impairment and severe dementia, characterized as inability or showing difficulty to communicate. A written informed consent was obtained from all participants’ caretakers (parents or legal surrogates). The Consort Guidelines for reporting Randomized Controlled Trials were followed for the presentation of the study.

Fifty-seven consecutive DS patients were assessed and those found eligible for inclusion were randomly assigned to receive either desflurane (DES-group, n = 22) or sevoflurane (SEVO-group, n = 22) for maintenance of anesthesia, according to a computer generated list. Randomization was performed using the study randomization engine created by Urbaniak, G. C., & Plous, S. in 2013 [Research Randomizer (Version 4.0) (Computer software, retrieved on June 22, 2013, from http://www.randomizer.org/)].

The primary outcome measure was the early postoperative cognitive function, as assessed with the Prudhoe Cognitive Function Test (PCFT) which is designed to “quantitatively” measure cognitive function in people with any degree of ID. It was initially designed to establish a baseline of pre-existing cognitive functioning in adults with DS [15]. It assesses competencies in five areas, Orientation (checking if the participant is orientated temporally - comparing events in relationship to when they occur), Recall (checking memory skills), Language (a test of verbal expression - not of knowledge), Praxis (assessing ability of the participant to demonstrate adequately how to carry out the appropriate actions) and Calculation (counting) [15–17]. It is simple and takes a maximum of 20 min to complete. It consists of 84 items and has a maximum overall score of 240 points. A score below 128 indicates a severe ID [15]. It is sensitive in identifying cognitive decline over time and this is the reason it was considered appropriate for the aim of this study, dealing with cognitive changes over time perioperatively. Patients’ screening and the test were performed in the hospital ward. The environment for the test was kept quiet with only one familiar person to the patient in the room and removal of any stimuli that could be distracting.

In the operating room a 20-gauge intravenous (IV) cannula was inserted for fluid and drug administration. Monitoring included electrocardiogram, pulse oximetry, noninvasive blood pressure, capnography and neuromuscular transmission (NMT) monitoring (TOF-Scan®; IDMed; Marseille, France). Prior to anesthesia induction, ondansetron 4 mg, metoclopramide 10 mg, dexamethasone 4 mg for prevention of postoperative nausea/vomiting (PONV) and paracetamol 1000 mg for pre-emptive analgesia were administered IV. General anesthesia was induced with fentanyl 50 mcg, propofol 2.5 mg/kg and rocuronium 0.9 mg/kg for facilitation of tracheal intubation. Anesthesia was maintained with desflurane (DES-group) or sevoflurane (SEVO-group) in an oxygen/air mixture (FiO_2_: 0.4, total fresh gas flow rate: 1.5 L/min). The administered volatiles were adjusted to target a minimum alveolar concentration (MAC) of 1, modified for age. Ventilation was controlled to maintain normocarbia (End tidal CO2, ETCO2: 35–40 mmHg).

At the end of surgery, following the last surgical manipulation, the volatile agent was discontinued, total fresh gas flow rate was increased to 8 L/min, FiO_2_ was increased to 1.0 and residual neuromuscular blockade was reversed with sugammadex. Extubation was attempted when the NMT’s train-of-four *(*TOF) ratio was 0.9 and sufficient spontaneous breathing in an awake patient was achieved.

Afterwards, all patients were transferred to the post-anesthesia care unit (PACU), where standard monitoring was applied. Patients were discharged from the PACU to the ward with a modified Aldrete score of 9 or 10 [18]. In case post-operative analgesia was required, parecoxib 40 mg was administered. Ondansetron 4 mg was used to treat PONV. Patients were considered ready to be discharged home when they had a Post-Anesthesia Discharge Scoring System (PADSS) score of 9 or more [19]. Τhe PADSS is used for outpatients and includes the following 6 criteria, which are scored from 0 to 2: vital signs, ambulation, PONV, pain, bleeding and voiding. A score of 9 or 10 is considered safe for outpatients to be discharged from hospital [19].

During anesthesia, the following data were recorded: systolic, diastolic, and mean arterial pressure (SAP, DAP, and MAP, respectively), heart rate (HR), ETCO_2_, hemoglobin saturation (SpO_2_), inhaled and exhaled volatile anesthetic concentration and MAC values.

The secondary outcome measures were related to recovery characteristics. We measured the time between discontinuation of the volatile agent and first spontaneous breath, first eye opening, extubation, orientation in place and first responding to verbal commands. Also, the time of fulfillment of the criteria to discharge from PACU (Aldrete score ≥ 9), orientation in place, time and person in the PACU, postoperative need for antiemetics and analgesics were recorded. Additionally, the caretaker was asked to assess him/herself the patient’s alertness (1 point for sleepy, 2 points for tired, 3 points for awake), wellness (1 point for poor, 2 points for moderate, 3 points for good, 4 points for excellent) and energy (1 point for poor, 2 points for moderate, 3 points for normal), preoperatively and also at 90 min and 4 h postoperatively. Satisfaction from anesthetic handling reported by the caretakers was recorded as well, using a 0 to 10 scale (0 for the worst and 10 for the highest possible satisfaction). Finally, the time of fulfillment of the criteria of PADSS were recorded.

The study is double-blind as neither the patients and caretakers nor the investigators who were involved in the primary and secondary outcome measures (PCFT and recovery characteristics) where aware of the patient group allocation. Regarding recovery characteristics recorded in the operating theatre, a blinded observer was entering the room after discontinuation of the volatile agent and after covering the relevant area on the monitor.

### Statistical analysis

The methodology of power analysis represented a design, with two levels of the between-subject factor of the two study groups and three levels of the within-subjects factor of time. A repeated measures analysis of variance (ANOVA) power analysis was conducted. The power calculation was performed a-priori, based on effect sizes of mean differences that we could detect at every quantitative measure of the study. The effect size for this calculation used the ratio of the standard deviation of the effects for a particular factor or interaction and the standard deviation of within-subject effects. The power analysis was conducted for a single, two-group between-subjects factor, and a single within-subjects factor assessed over three time points. For this design, 44 participants (22 per group) achieves a power of 0.90 for the within-subjects main effect at an effect size of 0.20; a power of 0.90 for the between-subjects main effect at an effect size of 0.36; and a power of 0.90 for the interaction effect at an effect size of 0.22.

Continuous variables are presented with mean and standard deviation (SD) and/or with median and interquartile range (IQR). Quantitative variables are presented with absolute and relative frequencies. For the comparison of proportions chi-square tests were used. For the comparison of continuous variables between the two study groups, the Student’s t-test was computed in case of normal distribution and the Mann-Whitney test in case of not normal distribution. Differences in changes of PCFT, Alertness, Wellness and Patient Energy during the follow up period between the two study groups were evaluated using repeated measurements ANOVA. Variables that had skewed distribution were log-transformed for the analysis of variance. All *p* values reported are two-tailed. Statistical significance was set at 0.05 and analyses were conducted using SPSS statistical software (version 22.0).

## Results

**Fig. 1:**
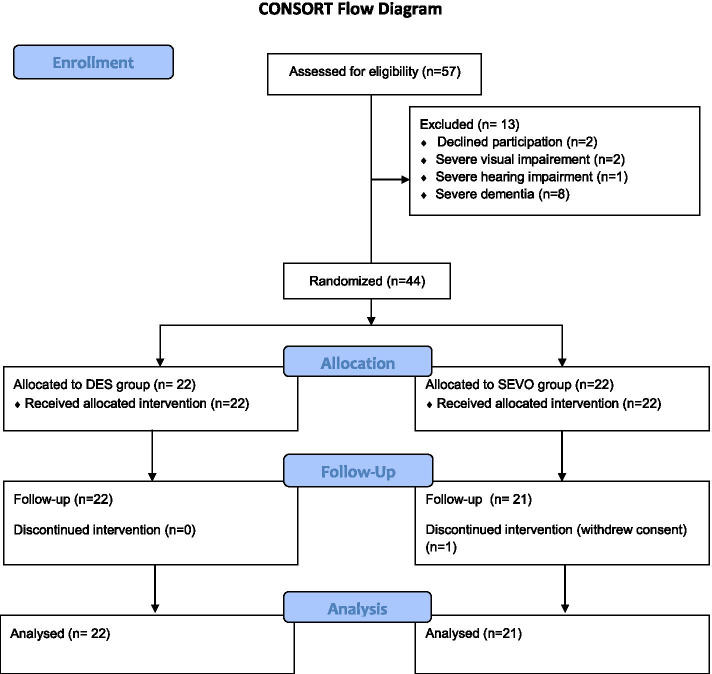
Consort flowchart of the study

Data from 43 patients (22 in the DES-group, 21 in the SEVO-group) were analyzed, in a 24 months period, as shown in the flow diagram of the study (Fig. 1). The demographic and operative characteristics of the patients were similar between the two groups (Table 1).

**Table 1:** Patient and operative characteristics by study group (Desflurane group: DES-group and Sevoflurane group: SEVO-group)

	DES-group, N = 22	SEVO-group, N = 21	*P*-value
Male gender, N (%)	13 (59.1)	13 (61.9)	0.850^++^
Female gender, N (%)	9 (40.9)	8 (38.1)	
Age, mean (SD)	34.5 (10.4)	29.1 (10.8)	0.102^+^
BMI, mean (SD)	24.9 (3.11)	23.5 (3.73)	0.19^+^
ASA I, N (%)	7 (31.8)	7 (33.3)	0.916^++^
ASA II, N (%)	15 (68.2)	14 (66.7)	
Duration of surgery (min), mean (SD)	71.2 (24.9)	62.7 (17)	0.200^+^
Duration of anesthesia – induction to extubation (min), mean (SD)	96.4 (27.1)	87.3 (18.1)	0.204^+^
Mean arterial pressure (mmHg), mean (SD)	73.2 (10.9)	75.7 (10.5)	0.224^+^
Heart rate (beats per minute), mean (SD)	74.9 (12)	74.3 (11)	0.671^+^
Mean MAC, mean (SD)	0.92 (0.1)	0.89 (0.2)	0.120^+^

^+^Student’s t-test; ^++^Pearson’s chi-square test; *ASA* American Society of Anesthesiologists

The recorded PCFT scores of the study groups during the follow up period (preoperatively-baseline, at 90 min and at 4 h) are presented in Table 2. The baseline of the total PCFT scores -along with its five domains individually (Orientation, Recall, Language, Praxis and Calculation)- did not differ statistically between the DES and SEVO-group. Intragroup comparisons showed that 90 min after recovery, total PCFT scores, Orientation, Language, Praxis and Calculation domain scores decreased significantly from baseline in both groups. Recall decreased significantly only in the SEVO-group. Intergroup comparisons showed that total PCFT scores and Orientation, Language, Praxis and Calculation domain scores were significantly higher in the DES versus SEVO-group (*p* < 0.05). Four (4) hours after recovery, total PCFT scores, Orientation, Recall, Language and Praxis domain scores reached levels similar to baseline scores in the DES-group, while in the SEVO-group they remained significantly lower. The scores in the Calculation domain did not differ significantly between the two groups. The overall change of total PCFT scores and all domains with the exception of Orientation and Calculation, during the follow up was significantly different between the two groups as indicated from the significant interaction effect of the analyses (P_3_ in Table 2).

**Table 2:** Changes in PCFT during the follow up for the two study groups (Desflurane group: DES-group and Sevoflurane group: SEVO-group)

	Group	Baseline	90′ after recovery	4 h after recovery	P_2_ Baseline vs 90′	P_2_ Baseline vs 4 h	P_2_ 90′ vs 4 h	P_3_
PCFT total scores	DES	137 (54.6)	125.6 (52.5)	133.4 (53.1)	<0.001*	0.163	<0.001*	<0.001*
	SEVO	111.57 (55.47)	78.76 (52.41)	94 (53.97)	<0.001*	<0.001*	<0.001*	
	P_1_	0.138	0.006*	0.021*				
Orientation	DES	11.7 (4.9)	10.5 (4.9)	11.6 (5)	0.033*	1.000*	<0.001*	0.073
	SEVO	8.96 (3.82)	6.67 (3.25)	7.95 (3.2)	<0.001*	0.018*	<0.001*	
	P_1_	0.054	0.004*	0.006*				
Recall	DES	10.3 (5.4)	10 (5.5)	10.4 (5.3)	1.000	1.000	0.986	0.033*
	SEVO	10.67 (4.62)	7.52 (4.09)	9.14 (3.88)	<0.001*	0.017*	<0.001*	
	P_1_	0.799	0.102	0.397				
Language	DES	59.5 (20)	54.6 (20.7)	58.6 (20.9)	0.005*	1.000	0.003*	0.001*
	SEVO	48.19 (23.53)	35.14 (26.27)	41.19 (25.1)	<0.001*	<0.001*	<0.001*	
	P_1_	0.098	0.010*	0.018*				
Praxis	Group	Baseline	90′ after recovery	4 h after recovery	P_2_ Baseline vs 90′	P_2_ Baseline vs 4 h	P_2_ 90′ vs 4 h	P_3_
	DES	41.1 (18.9)	36.9 (16.6)	39.9 (17.9)	0.016*	0.521	0.012*	<0.001*
	SEVO	34.19 (21.8)	22.48 (17.53)	27.62 (19.14)	<0.001*	<0.001*	<0.001*	
	P_1_	0.273	0.008*	0.036*				
Calculation	DES	14.4 (12.7)	12.4 (10.7)	12.9 (10.6)	0.034*	0.056	0.656	0.646
	SEVO	9.19 (7.48)	6.95 (6.05)	8.1 (6.46)	0.021*	0.263	0.043*	
	P_1_	0.110	0.049*	0.082				

*PCFT* Prudhoe Cognitive Function Test. Data are presented as mean (SD); * indicates statistical significance (*P* < 0.05); P_1_: *P*-value for group effect; P_2_: *P*-value for time effect; P_3_: Effects reported include differences between the groups in the degree of change (repeated measurements ANOVA)

Patients’ alertness, wellness and energy were restored to their preoperative values at 90 min in the DES-group while in the SEVO-group they remained significantly reduced compared to baseline. In the SEVO-group alertness and wellness were restored at 4 h, but energy remained reduced, even though the statistical significance was marginal (*p* = 0.049 for comparison with baseline), as shown in Table 3.

**Table 3:** Changes in Patient Alertness, Wellness and Energy during the follow up for the two study groups (Desflurane group: DES-group and Sevoflurane group: SEVO-group)

	Group	Baseline	90′ after recovery	4 h after recovery	P1-value Baseline vs 90′	P2-value Baseline vs 4 h	P3-value 90′ vs 4 h
Patient Alertness	DES	2.91 (0.29)	2.82 (0.39)	2.91 (0.29)	1.000	1.000	1.000
	SEVO	2.62 (0.5)	2.1 (0.54)	2.43 (0.51)	<0.001*	0.220	0.019*
Patient Wellness	DES	2.73 (0.46)	2.32 (0.65)	2.73 (0.55)	0.080	1.000	0.084
	SEVO	2.29 (0.78)	1.38 (0.59)	2.05 (0.74)	<0.001*	0.375	<0.001*
Patient Energy	DES	2.82 (0.39)	2.64 (0.49)	2.86 (0.35)	0.329	1.000	0.200
	SEVO	2.86 (0.36)	2 (0.45)	2.57 (0.51)	<0.001*	0.049*	<0.001*

All comparisons were made using logarithmic transformations; Data are presented as mean (SD); * indicates statistical significance (*P* < 0.05); P_1_: *p*-value for group effect; P_2_: *p*-value for time effect; P_3_: Effects reported include differences between the groups in the degree of change (repeated measurements ANOVA)

Time to first eye opening, time to orientation in place, time to first responding to verbal commands, time to reach modified Aldrete score ≥ 9 in PACU and time to fulfill discharge criteria (PADSS) were significantly shorter in the DES-group as compared to SEVO-group. Time to first spontaneous breath and extubation did not differ significantly between the groups (Table 4). No additional need for antiemetics and analgesics was recorded.

**Table 4:** Comparison of Recovery characteristics between the two groups (Desflurane group: DES-group and Sevoflurane group: SEVO-group)

	DES-group	SEVO-group	*P*-value ^+^
Time to first spontaneous breath (min)	13.5 (4.3)	15.7 (5.1)	0.122
Time to open eyes (min)	14.8 (4.9)	18.6 (5.3)	0.021*
Time to extubation (min)	16.2 (4.7)	19 (5.9)	0.085
Time to spatial orientation (min)	19.3 (5.2)	25.7 (8.3)	0.004*
Time to respond to commands (min)	15.8 (4.9)	21.4 (7.2)	0.004*
Time to reach Aldrete score ≥ 9 in PACU (min)	19 (4)	25.7 (12.2)	0.018*
Time to fulfill PADSS (min)	221.8 (25.6)	265 (35.3)	<0.001*

Data are presented as mean (SD); ^+^indicates the use of Student’s t-test; * indicates statistical significance (*P* < 0.05);

Regarding intraoperative parameters, there was no statistically significant difference between the groups (*p* > 0.05) in baseline values, overall mean SAP, DAP and MAP. Mixed-effects linear regression analysis to check if measurements changed differently between groups, showed no significant change in SAP, DAP and MAP in either group. There was no statistically significant difference between groups in baseline HR and overall mean HR (*p* > 0.05). Mixed-effects linear regression analysis showed a significant decrease in HR during measurement period in both groups, which was less in the DES-group. There was no statistically significant difference between groups in baseline and overall mean SpO_2_ and ETCO_2_ (*p* > 0.05).

Finally, desflurane was associated with a superior overall patient experience. Satisfaction score in the DES-group had a mean equal to 9.8 (SD = 0.5) and median 10 (IQR:10–10), while satisfaction score in the SEVO-group was significantly lower, with a mean equal to 9.1 (SD = 1.2) and median 9 (IQR:9–10), (Mann Whitney test, *p* = 0.008).

## Discussion

The present study investigated the impact of the two most popular volatiles, desflurane and sevoflurane, on postoperative cognitive function and recovery characteristics in DS population. Regarding the primary outcome, our results demonstrated that early postoperative cognitive function scores were higher when anesthesia was maintained with desflurane. The difference was more apparent 90 min after recovery, but remained significant for at least 4 h. Most importantly, 4 h after recovery, there was no significant change from baseline in DES-group while in SEVO-group the difference from the preoperative baseline remained statistically significant. Desflurane was also found superior in terms of recovery times. Finally, the discharge criteria from both PACU and hospital were fulfilled earlier when desflurane was used. The favorable recovery characteristics of desflurane identified by our study can be mainly attributed to its low solubility in blood and body tissues (blood: gas partition coefficient of 0.42 and fat: blood solubility 27 at 37 °C) compared to sevoflurane (blood: gas partition coefficient of 0.65 and fat: blood solubility 48 at 37 °C) [20].

Various studies have investigated the impact of anesthetic agents on recovery and postoperative cognition, but it is difficult to compare our findings to theirs, since most of them were performed in elderly patient populations and have different methodologies, mainly using the Mini-Mental State Examination (MMSE) [5–14]. Additionally, they mostly focus on postoperative delirium in older age groups and postoperative cognitive dysfunction (POCD) that appears after patient discharge. Among these studies, those which focused on recovery characteristics, suggest that desflurane is superior [6, 8, 13] or comparable to sevoflurane [10, 11]. Similarly, studies focused on cognitive function, find desflurane slightly superior [7, 8, 12] or equivalent to sevoflurane [5, 9, 10, 14]. To our knowledge there are no studies investigating early recovery and cognitive characteristics in younger patients with DS or any intellectual disability.

On a first read, a difference in the PCFT scores between the two anesthetic groups at 90 min and 4 h after surgery, as well as the recovery characteristics, could seem unlikely to be clinically significant. On the contrary, we consider that in this patient population these parameters may be of major clinical significance. In the PACU or other recovery units, patients with intellectual disabilities show several problems. The personnel in these units are usually limited and have to take care of more than one patient. This could lead to deficits in the special care that most DS patients might need, since close observation is essential until they are fully recovered from anesthesia, and even afterwards. Moreover, patients with ID are also at higher risk for inadequate analgesia due to their limited capacity to express verbally or behaviorally their pain or other unpleasant feelings. Occasionally they feel excessive discomfort. Unrelieved pain, stress and fear may further exacerbate their cognitive impairment [21]. Their limited communication skills and the presence of maladaptive behaviors may cause distress to them and their caretakers [22–25]. Thus, it is imperative to use anesthetic techniques that may ensure the earliest return of cognitive function possible, so that this sensitive patient population may express their feelings and get adequate relief of their pain, distress and fear.

Patients’ alertness, wellness and energy were assessed by the caretakers. This could render assessment subjective but on the contrary, these results provide more valuable and accurate data regarding caretakers’ satisfaction grade and confidence to the anesthetic technique and patient safety. Higher scores mean that caretakers feel more comfortable and sure that fast-tracking will not jeopardize patients’ safety.

The use of appropriate drugs and anesthetic techniques may allow the phase of early recovery to be complete before the transfer to PACU, and some patients can bypass the first stage recovery (patient is awake, protective airway reflexes have returned, pain is controlled) [4, 26]. The second-stage recovery follows, ending when patients are ready for hospital discharge [4]. Even mild postoperative cognitive dysfunction and discomfort may delay this stage and prolong hospital stay. The latter may have a negative impact on patients’ and caretakers’ satisfaction, but most importantly it may increase the risk of hospital-acquired infections, especially in DS patients who are more susceptible due to dysfunction of the cellular and humoral immunity [4, 27]. There is a major effort worldwide to promote day surgery and despite the fact that guidelines are published, ongoing studies explore techniques and pathways to ensure that day surgery is applied to more patient groups. The need to minimize length of stay and improve the quality of postoperative recovery have ensured that day surgery principles are fundamental to modern patient care. Our study suggests that desflurane fits better as a maintenance agent for these cases.

One of the main strengths of our study is the homogeneity of the sample, that is DS patients with similar phenotypes and pathophysiology of ID. It was challenging and time consuming to gather the required number of unique DS patients. Another advantage is the use of PCFT test which has been specifically constructed to assess the cognitive function in patients with ID of any degree. It is simple and can be carried out by non-specialist evaluators. It is considered highly reliable, since it is given directly to the individual concerned and is not based on information provided by others [15, 16].

There are some limitations to this study. Patients were followed up for the study outcomes until they were discharged from hospital and not afterwards. Nevertheless the caretakers were instructed to call in case of any complication or question. There was no any phone call recorded. Also, they were not screened for the development of delirium, that is an outcome measure of several studies. But even if we did not use a delirium-dedicated scale like Nursing Delirium Screening Scale (NuDESC) or Confusion Assessment Method (CAM), delirium could be suspected indirectly through the patients’ performance on the PCFT test. We did not suspect such a case. Moreover, the target age group of delirium is much older than our sample’s age. Αnother point to note is that individuals with DS present ID of variable degrees, thus affecting the baseline PCFT scores. Νevertheless, the baseline mean values did not differ between groups, while data analysis focused on the change from the baseline and not the actual score numbers in order to overcome the variability in the degree of ID.

## Conclusions

Under the present study design, desflurane was found superior to sevoflurane in terms of faster recovery and better preserved postoperative cognitive function in DS patients undergoing dental surgery. Thus, we suggest that desflurane, as part of a multimodal anesthetic approach, could be a useful agent to enhance fast-tracking and early discharge from hospital of ambulatory patients with ID.

## Data Availability

The datasets used and/or analyzed during the current study available from the corresponding author on reasonable request.

## Abbreviations

ANOVA: Analysis of variance
ASA: American Society of Anesthesiologists
CAM: Confusion Assessment Method
DAP: Diastolic arterial pressure
DS: Down syndrome
HR: Heart rate
ID: Intellectual disability
IQR: Interquartile range
IV: Intravenous
MAC: Minimum alveolar concentration
MAP: Mean arterial pressure
MMSE: Mini-Mental State Examination
NMT: Neuromuscular transmission
NuDESC: Nursing Delirium Screening Scale
PACU: Post-anesthesia care unit
PADSS: Post-Anesthesia Discharge Scoring System
PCFT: Prudhoe Cognitive Function Test
POCD: Postoperative cognitive dysfunction
PONV: postoperative nausea/vomiting
SAP: Systolic arterial pressure
SD: Standard deviation
SpO_2_: Hemoglobin saturation
TOF: Train-of-four
